# GC/MS-Based Metabolomics Approach to Evaluate the Effect of Jackyakgamcho-Tang on Acute Colitis

**DOI:** 10.1155/2019/4572764

**Published:** 2019-01-21

**Authors:** Seung-Ho Seo, Seong-Eun Park, Eun-Ju Kim, Daehwan Youn, Yu-Mi Lee, Soon-Young Lee, So-Hyeon Bok, Dae-Hun Park, Chang-Seob Seo, Sung-Hoon Byun, Ki Young Jun, Dae Sung Kim, Chang-Su Na, Hong-Seok Son

**Affiliations:** ^1^School of Korean Medicine, Dongshin University, Naju 58245, Republic of Korea; ^2^Herbal Medicine Research Division, Korea Institute of Oriental Medicine, Daejeon 34054, Republic of Korea; ^3^Department of Oral and Maxillofacial Surgery, Gyeongsang National University Hospital, Jinju 52727, Republic of Korea; ^4^Hanpoong Pharm. Co., Ltd., Wanju 55316, Republic of Korea

## Abstract

The objective of this study was to examine the effects of Jackyakgamcho-tang (JGT) on acute colitis. GC/MS-based metabolomics and NGS-based metagenomics were applied to investigate the alteration of metabolites and microbiota in an acute colitis model. The severity of acute colitis symptoms was alleviated by JGT treatment. Induction of colitis and JGT treatment changed compositions of gut microbiota and inflammatory cytokine levels (TNF-*α* and IL-6). They also substantially change metabolites (i.e., lactic acid, linoleic acid, monostearin, and palmitoylglycerol). In addition, some clear correlations were observed among metabolites, cytokine, and microbiota. This study highlights the applicability of metabolomics and metagenomics study for evaluating anti-inflammatory effects of a new functional herbal medicine as a therapeutic agent for acute colitis.

## 1. Introduction

Inflammatory bowel disease (IBD) is a multifactorial disease that is difficult to identify the cause of onset [1]. Clinical features of IBD include abdominal pain, hemorrhagic diarrhea, and weight loss [2]. Some drugs such as 5-aminosalicylate and corticosteroid are used in the treatment of IBD, but long-term use of these drugs reduces efficacy and causes side effects. Recently, gut microbiome profiling studies have revealed that the occurrence and recovery of IBD are associated with changes of gut microbiota [3]. It has been also reported that gut microbiome is involved in the regulation of the host's metabolism. For example, nondigestible carbohydrates are fermented by gut bacteria to produce metabolites that can act as inflammatory modulators and signaling molecules [4].

Treatment of IBD by natural products (herbal medicine) can increase treatment effects with reduced side effects [5]. Substances such as andrographolide [6], andrographolide sulfonate [7], baicalin [8], curcumin [9], and baicalein [10] in natural products exhibit efficacy in treating IBD. Jackyakgamcho-tang (JGT) consists of* Glycyrrhiza uralensis* and* Paeonia lactiflora.* It is a Korean traditional herbal drug known to be effective for pain accompanied by muscle spasms. It contains various bioactive components with antioxidative, anti-inflammatory, antiviral, and neuroprotective effects [11, 12]. In Korean medicine, it is often prescribed for abdominal pain. However, effects of JGT against IBD-related recovery have not been fully studied [13–15].

Metabolomics involves comprehensive analysis of small-molecule metabolome within an organism or biological system [16]. Integral and systematic studies of metabolomics approaches are expected to provide new research methods for the study of Korean medicine. The purpose of this study was to examine the role of JGT in healing acute colitis. Next generation sequencing-based metagenomics and GC/MS-based metabolomics were employed in a profiling mode to reveal changes in microbial community structure and key metabolites.

## 2. Materials and Methods

### 2.1. HPLC Analysis of Major Components in JGT

A quantitative analysis of ten major compounds in JGT was performed with a Prominence LC-20A (Shimadzu, Kyoto, Japan) equipped with a photo-diode array detector. The marker components were separated on a Phenomenex Gemini C18 column (250 × 4.6 mm, 5 *μ*m, Torrance, CA, USA) at column oven temperature of 40°C. The mobile phase consisted of 0.1% (v/v) aqueous formic acid and acetonitrile. Gradient flow was as follows: 8–55% B for 0–40 min, 55-78% B for 40-43 min, 78% B for 43-46 min, and 78-8% B for 46-50 min. The flow rate was at 1.0 mL/min. Chemical structures of these 10 reference standard compounds for JGT analysis are provided in Figure S1.

### 2.2. Animals

Male Sprague Dawley rats weighing approximately 240-250 g were obtained from Samtako Bio Korea (Osan, Korea). These rats were kept in standard laboratory conditions with a room temperature of 24 ± 1°C, humidity of 65 ± 5%, and a controlled light/dark cycle (12/12 h). The rat experiments in the present study were performed in compliance with the guidelines of the Ethics Committee of Dongshin University with approval number of 2017-05-02. Rats were randomly assigned to four groups (*n *= 6/group): (1) vehicle control, (2) acetic acid-induced colitis, (3) colitis induced plus treatment with JGT at 150 mg/kg, and (4) colitis induced plus treatment with JGT at 300 mg/kg.

### 2.3. Induction of Acute Colitis and JGT Treatment

Acute colitis was induced according to the sequence described by Ghasemi-Pirbaluti et al. [1]. Briefly, rats were fasted for 24 h before colitis induction with ad libitum access to water. For acute colitis induction, 1 mL of acetic acid (3% v/v in normal saline) was infused into the rectum with an 8-cm long tube under anesthesia using 2.5% of isoflurane. The rats were positioned head-down for 30 s to avoid expelling the solution. JGT treatment was administered through oral intake of 150 mg/kg (JGT 150) or 300 mg/kg (JGT 300) of JGT in drinking water. JGT was treated for 3 days from the colitis induction day and the first day was administered 4 hours after colitis induction. These doses of JGT were chosen based on existing clinical dose (Hanpoong Pharmaceutical Company, Seoul, Korea). The quality control data about JGT was provided in Table S1. The vehicle control group was treated with 1 mL of saline. All experimental groups were provided free access to laboratory chow and water for 3 days. The samples for the analyses were obtained at 3 days after the induction of colitis. The detailed experimental design is illustrated in Figure S2.

### 2.4. Histopathological Analysis

Distal portions of the colon were embedded in paraffin, sliced (6-*μ*m in thickness), and stained with hemotoxylin and eosin (H&E). Inflammation and histological damage were observed under a light microscope (Nikon 80i, Japan) in a blinded manner.

### 2.5. Cytokine Quantification

Serum concentrations of cytokines TNF-*α* and IL-6 as indicators of inflammation were measured by ELISA kit (Invitrogen, USA). ELISA plates were read at 450 nm using a SpectraMax plate reader (M2, Molecular Devices, USA).

To measure the cDNA level of several cytokines such as IFN-*γ*, TNF-*α*, and IL-6, total RNA was extracted from the intestine using the RNeasy Mini Kit (Qiagen, Hilden, Germany) and 100 ng RNA was used for the reaction. The RT-PCR cycles consisted of denaturation at 95°C for 5 s and annealing/extension at 65°C for 30 s for 40 cycles.

### 2.6. Analyses of Microbiota in Feces

Metagenomic DNA was extracted from each feces sample of rats using a PowerSoil® DNA Isolation kit (Cat. No. 12888, MO BIO). The concentration of obtained DNA and DNA quality were assessed using PicoGreen and Nanodrop, respectively. After checking the quality of metagenomic DNA by gel electrophoresis, 16S rRNA genes were amplified using 16S V3-V4 primers. DNA sequencing was then performed using MiSeq platform (Illumina, San Diego, CA, USA). Data were processed using Quantitative Insights Into Microbial Ecology (QIIME) v1.8 analysis pipeline [17].

### 2.7. Metabolic Analysis

Freeze dried serum and feces samples (100 mg) were ultrasonicated with 300 *μ*L of solvent [methanol:water (7:3)] at 4°C for 30 min. Then, samples were centrifuged and lyophilized for derivatization. Sample derivatization and GC/MS analysis protocols were the same as described in our previous study [18].

### 2.8. Data Processing and Multivariate Analyses

GC/MS data were pretreated using XCMS web software (https://xcmsonline.scripps.edu) for noise removal, baseline correction, and alignment. Feature intensities were normalized according to the intensity of methyl stearate (internal standard) prior to multivariate statistical analyses. GC/MS preprocessing data files were imported into SIMCA-P (ver. 14.0) software package (Umetrics, Umea, Sweden) for multivariate analyses

## 3. Results

### 3.1. HPLC Analysis of JGT

Optimized HPLC-PDA method was used for quantitative determination of the ten major compounds in JGT composed of* Paeonia lactiflora* and* Glycyrrhiza uralensis*. As a result, these compounds (gallic acid, oxypaeoniflorin, albiflorin, paeoniflorin, liquiritin, benzoic acid, lsoliquiritin, ononin, benzoylpaeoniflorin, and glycyrrhizin) were eluted within 40 min (5.10, 11.35, 14.84, 15.88, 18.45, 21.49, 23.11, 24.08, 29.73, and 39.31 min, respectively). Representative HPLC-PDA chromatograms of the standard mixture and JGT sample are displayed in Figure S3. Based on the above results, amounts of these 10 compounds in JGT ranged from 0.34 mg/g to 30.94 mg/g (Table S2).

### 3.2. Clinical Symptoms

**Figure 1:**
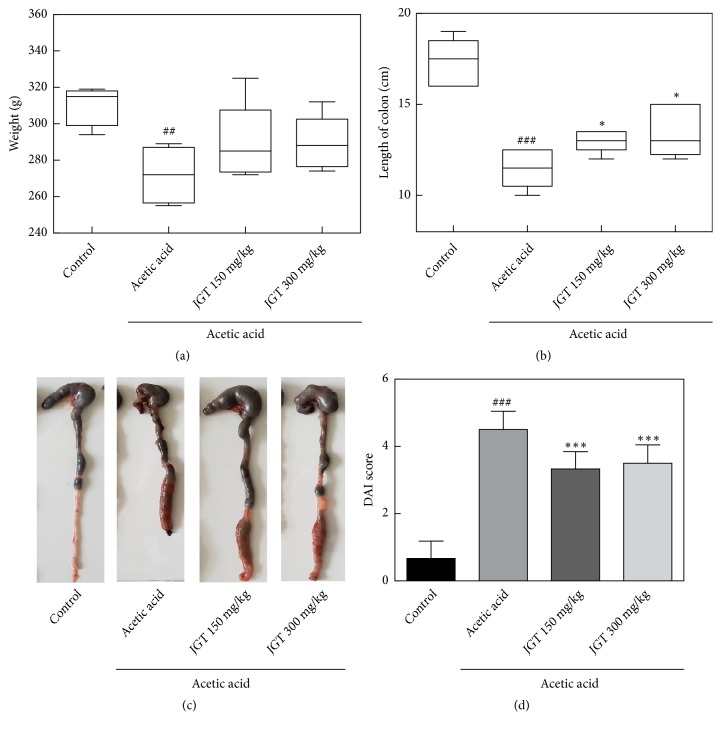
(a) Weight, (b) colon length, and (c) colon tissue pictures of rats treated with acetic acid and Jackyakgamcho-tang (JGT). (d) Disease activity index (DAI) value was calculated by using body weight loss, stool consistency, and blood in stool scores. Significant difference at  ^#^*p* < 0.05,  ^##^*p* < 0.01, and  ^###^*p* < 0.001 compared to the control group. Significant difference at  ^*∗*^*p* < 0.05,  ^*∗∗*^*p* < 0.01, and  ^*∗∗∗*^*p* <0.001 compared to acetic acid-induced acute colitis group.

**Figure 2:**
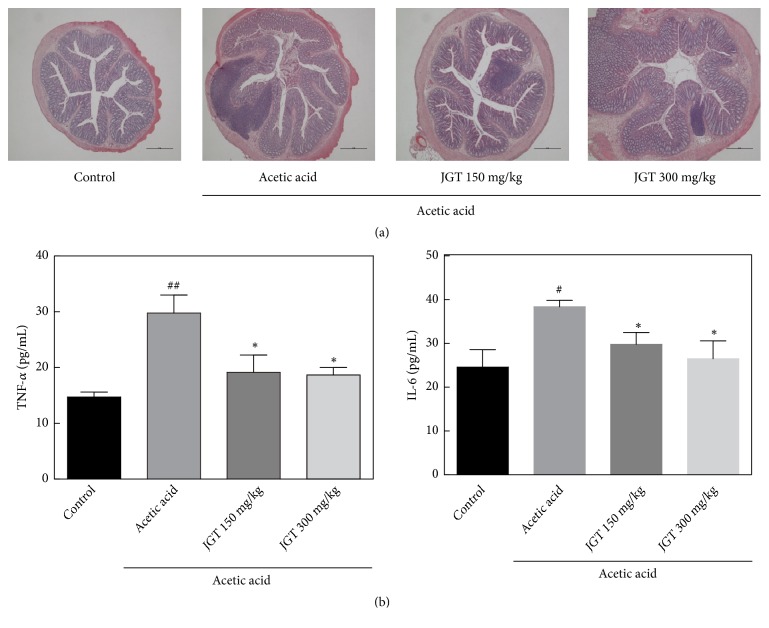
Effect of JGT on inflammation in acute colitis rat gut tissue. (a) Representative images by H&E staining displaying colon segments on the day of sacrifice. (b) Levels of tumor necrosis factor-*α* (TNF-*α*) and interleukin-6 (IL-6). Significant difference at  ^#^*p* < 0.05,  ^##^*p* < 0.01, and  ^###^*p* < 0.001 compared to the control group. Significant difference at  ^*∗*^*p* < 0.05,  ^*∗∗*^*p* < 0.01, and  ^*∗∗∗*^*p* <0.001 compared to acetic acid-induced acute colitis group.

To assess effects of JGT on acute colitis, changes in body weight, length of large intestine, and histopathological signs in colon tissues were determined (Figure 1). The weight of rats that had colitis induced by acetic acid was decreased on the 3rd day of colitis induction from their baseline body weight. Treatment with JGT slightly prevented such weight loss (Figure 1). Similarly, the symptoms of colitis were alleviated by JGT treatment. The shortening of colon length was found in colitis-induced group (12.3 ± 0.7 cm vs. 17.3 ± 0.6 cm,* p* < 0.05). Colon shortening is known to be positively associated with colonic inflammation from acute colitis [19]. Disease activity index (DAI) score was calculated by using body weight loss, stool consistency, and blood in stool scores. DAI score of the control group was 0.67. It was increased to 4.29 in colitis-induced group. Significant decrease in DAI score was observed in groups receiving either JGT 150 mg/kg (*p* < 0.001) or JGT 300 mg/kg (*p* < 0.01) when compared to colitis-induced group. Acetic acid-induced rats exhibited acute colitis with severe inflammation for histopathological examinations of colons (Figure 2(a)). However, treatment with JGT (150 mg/kg or 300 mg/kg) reduced cell infiltrate and crypt damage, indicating that JGT treatment could decrease the severity of acetic acid-induced acute colitis.

### 3.3. Inflammatory Cytokines

The pathogenesis of acute colitis is based on complicated cytokine-mediated signaling pathways [20]. Recent studies have demonstrated that most pathways are induced by intestinal T-cell activation via inflammatory mediators such as TNF-*α*, IL-1*β*, IL-6, IL-10, IL-12, and IL-23 [21–24]. Effects of JGT on cytokines' production are presented in Figure 2(b). Levels of TNF-*α* and IL-6 in the acute colitis group were noticeably higher than those in the control group. However, administration of JGT to colitis-induced rats significantly decreased levels TNF-*α* and IL-6, indicating reduced inflammation. Although some data are not significant, JGT administration slightly suppressed the levels of TNF-*α*, IL-6, and IFN-*γ* in the gut (Figure S4).

### 3.4. Metabolic Profiling

**Figure 3:**
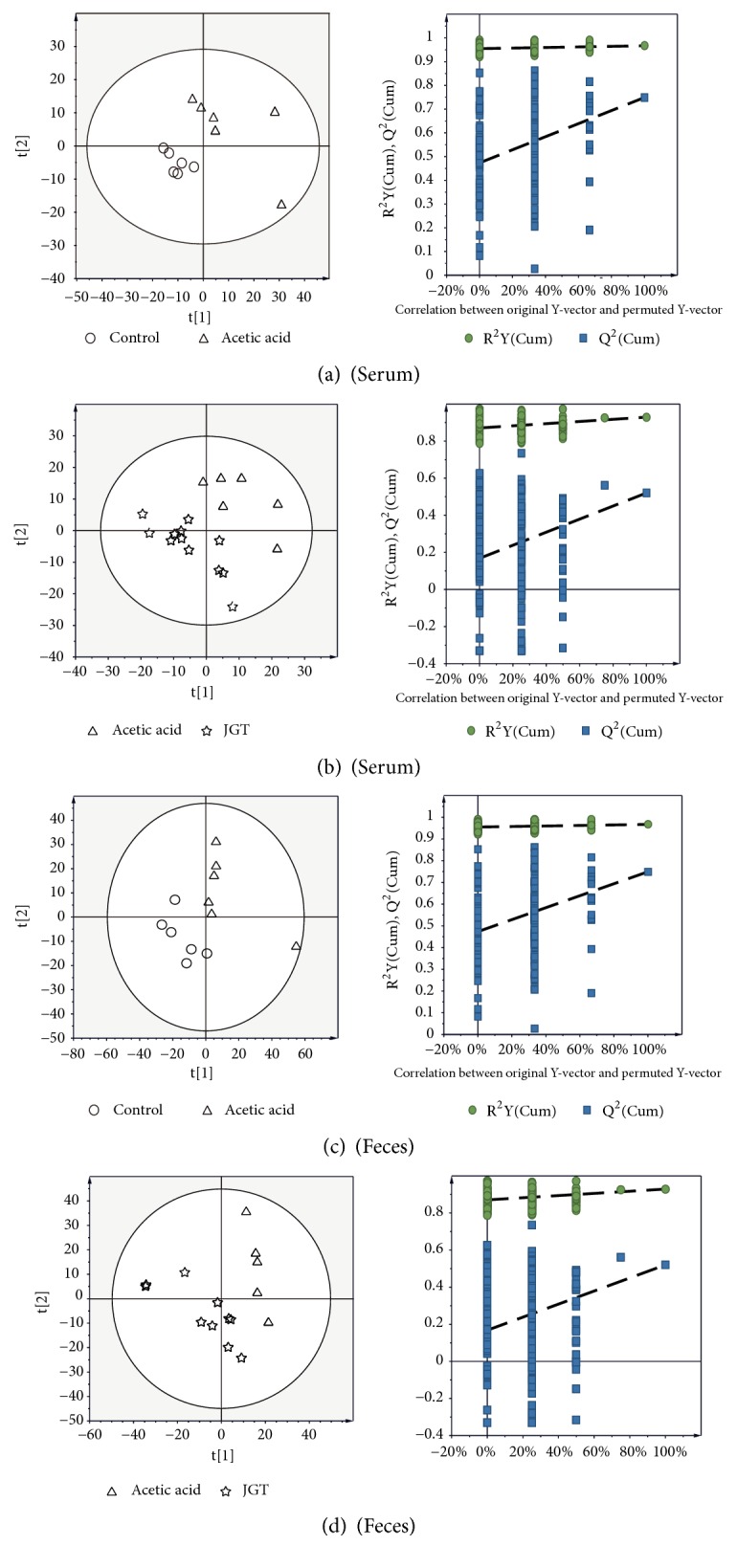
PLS-DA scores plots for control, acetic acid-induced acute colitis, and JGT treated groups derived from GC-MS data of serum (a, b) and feces (c, d) samples. These PLS-DA models were validated by a permutation test (n = 200).

Representative GC/MS total ion current chromatograms from these four groups are shown in Figure S5. A total of 29 metabolites were identified in the serum and feces samples based on RI value, GC/MS library, and other researchers' data (Table S3). A multivariate analysis was conducted using features and normalized mass intensity obtained from the GC/MS (Figure 3). Supervised PLS-DA was used to obtain a clear separation among groups [25].

PLS-DA score plots of serum and feces samples displayed a clear separation among groups, with high cumulative* R*^*2*^*X, R*^*2*^*Y, *and* Q*^*2*^ values. Low values of* Q*^*2*^ intercept in the permutation test indicated the robustness of models, demonstrating a low risk of overfitting [26]. These results indicated that metabolites of serum and feces could be changed by colitis induction and JGT treatment.

**Figure 4:**
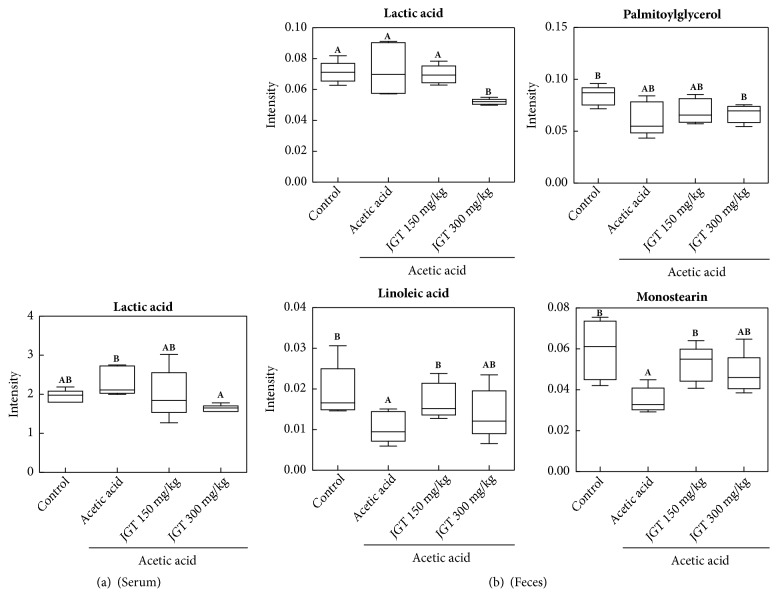
Box plots of identified metabolites that contributed to the discriminating PLS-DA model (VIP > 1,* p* < 0.05) in (a) serum and (b) feces. Peak intensities of selected mass ions from GC/MS data were used for quantification. Data were based on six replicates (n = 6). For each compound, the statistical outcome was summarized within the figure (one-way ANOVA). Alphabet represents significant difference between samples (*p* < 0.05).

To find metabolites responsible for the classification, the parameter of variable importance in projection (VIP) was determined. Based on VIP greater than 1.0 from PLS-DA with* p *< 0.05 in two-tailed Student's* t*-test, a total of four metabolites were identified as variables contributing to the separation of samples in the PLS-DA score plot (Figure 4 and Table S4). The acute colitis group was characterized by higher serum levels of lactic acid together with lower levels of linoleic acid, monostearin, and palmitoylglycerol in feces compared to the control group. However, samples obtained in the JGT treatment groups exhibited opposite metabolites patterns except for palmitoylglycerol compared to the acute colitis group.

### 3.5. Gut Microbiota Changes

A metagenomics approach was used to analyze diversity of the microbial community after colitis induction and treatment with JGT. To analyze microbial richness and diversity in feces samples according to colitis induction, rarefaction curves at 97% similarity levels were calculated. Microbial richness and diversity of 16S rRNA libraries are shown in Table S5. Chao 1 richness values of feces samples were 327.8, 299.9, 271.9, and 298.5 for control, acetic acid, JGT 150, and JGT 300 groups, respectively. Simpson's diversity values of feces samples were 0.94, 0.83, 0.92, and 0.74 for control, acetic acid, JGT 150, and JGT 300 groups, respectively. Operational taxonomic units (OTUs) in control samples were also higher than those in other samples, implying more bacterial diversities in feces of control rats. Microbial community analysis results are shown in Figure S6.* Bacteroidetes* (35.5%) and* Firmicutes* (33.2%) were the two most abundant phylums in feces of the control group. However,* Bacteroidetes* was less abundant in rats with colitis induction or treatment with JGT compared to that in the control sample.* Verrucomicrobia* became the main component in microbial communities of colitis-induced rats except for those that received JGT 150 treatment.

### 3.6. Correlations among Metabolites, Gut Microbiota, and Inflammatory Cytokines

**Figure 5:**
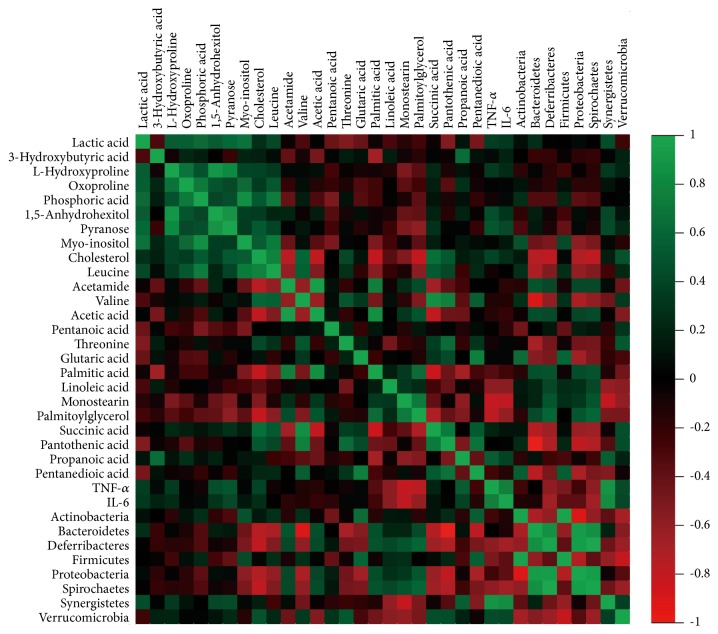
Integration of variable correlations. The heat map derived from correlations among metabolites of serum and feces, inflammatory cytokines, and gut microbiota phyla.

To explore functional relationships among altered gut microbiota, disturbed metabolites in serum and feces, and changed inflammatory cytokine levels (TNF-*α* and IL-6), three correlation matrixes were formed based on Pearson's correlation coefficients (Figure 5). Some variables were clearly correlated (r > 0.8 or r < -0.8). Hydroxyproline was positively correlated with 1, 5-anhydrohexitol and pyranose (r = 0.90 and r = 0.83, respectively). Acetic acid and valine were also positively correlated with palmitic acid (r = 0.85) and succinic acid (r = 0.85). Inflammatory cytokine levels (TNF-*α* and IL-6) displayed strong correlations with increased levels of* Synergistetes* (r = 0.88 and r = 0.84, respectively).

## 4. Discussion

Recently, the use of herbal remedies has emerged as an alternative treatment for colitis [27–29]. Li et al. [30] have reported that YunNan BaiYao (YNBY), an amalgamation of various herbs, can significantly decrease disease progression of DSS- and TNBS-induced colitis with reduced levels of inflammatory cytokines such as TNF-*α*, IL-12, IFN-*γ*, and IL-17 in the colon and serum. In another study, Huang et al. [29] have reported the anti-inflammatory effect of* Wedelia chinensis* extracts in a colitis mouse model. As a Korean traditional herbal medicine, JGT is composed of* Paeonia lactiflora* and* Glycyrrhiza uralensis *whose leaf and stem tissues are commonly used as traditional medicinal herbs in many Asian countries. It is known to be effective for pain accompanied by muscle spasms. In this study, various bioactive compounds, including gallic acid, paeoniflorin, and glycyrrhizin, were detected in JGT. These compounds have been reported to possess anti-inflammatory, antioxidant, and immune functions [31, 32]. Pandurangan et al. [12] have reported that the administration of gallic acid can significantly (*p* < 0.05) suppress expression levels of inflammatory cytokines in DSS-induced colitis mice. In this study, JGT was effective in protecting against acetic acid-induced acute colitis. Its inhibition on TNF-*α* and IL-6 expression was observed in serum of test rats treated with JGT (Figure 2(b)). The expression of TNF-*α* was highly correlated with the development of colitis [33]. Therefore, the antiacute colitis activity of JGT may be regulated by its inhibition effect on TNF-*α* expression.

In the current study, multivariate statistical approaches were used to analyze metabolites present in the serum and feces samples. The PLS-DA model revealed a clear separation between not only the control and acetic acid-induced colitis groups, but also acetic acid-induced colitis and JGT treatment groups. These results suggest that metabolites of the serum and feces can be changed by colitis induction and JGT treatment. The acute colitis group was characterized by higher levels of lactic acid in the serum compared to control and JGT treatment groups. Several studies have reported that the incidence of colitis is related to the change in lactic acid concentrations. Song et al. [34] have found that levels of lactic acid are increased in inflammatory intestinal mucosa of IBD patients. The damage of intestinal endotheliocytes can increase the permeation of lactic acid produced by microorganisms in the intestine. Vernia et al. [35] have reported that lactic acid level is increased in feces water of severe colitis subjects. Another mechanism of increased lactic acid in colitis may be caused by changes in microorganisms in the intestine. Increased intraluminal oxygen concentration due to profuse bleeding favors facultative anaerobic strains such as* lactobacilli* and* streptococci* known to be lactic acid producers [35, 36]. Increased concentrations of lactic acid may also result from disruption of colonic mucosal cell lining and mesenchymal polysaccharides exposure to intraluminal bacteria [36]. Lactic acid has been reported to accumulate in feces from individuals who are suffering from ulcerative colitis. Scharff et al. [37] reported that eighteen of the 42 ischemic colitis patients (42%) had an abnormally elevated serum lactic acid level. According to the findings of Gilshtein et al. [38], the only laboratory factor found as a significant risk factor for mortality was increased lactic acid levels.

There are controversial studies on the relationship between linoleic acid contents and colitis. Some studies have reported the contribution of linoleic acid to inflammation [39, 40]. On the contrary, linoleic acid has been reported to have potential to inhibit inflammatory responses by lowering the production of inflammatory cytokines such as TNF-*α* and IL-1*β* [41]. In addition, linoleic acids can be transformed into transfatty acids and conjugated linoleic acids (CLA) by microorganisms in the intestine [42–44]. Several studies have reported that CLA and conjugated linolenic acid (CLNA) can suppress colonic inflammation and upregulate colonic peroxisome proliferator-activated receptor (PPAR) *γ* expression [45–48].

A combination of metagenomics and metabolomics can improve our understanding of the human gut microbiome and possibly provide a new strategy for the diagnosis and treatment of disease. In this study, bacterial diversities in the feces of the colitis group were lower compared to those of the control. A disbalance of commensal microbiota with decreased diversity and altered metagenome and metabolome might be linked to colitis [49–51]. There are many reports on the relationship between colonic microbial community and metabolites. Weir et al. [52] have reported that a mucin-degrading species (*Akkermansia muciniphila*) is about 4-fold higher while butyrate-producing species are underrepresented in colorectal cancer samples, although there are no significant differences in overall gut microbiome. Gut microorganisms can synthesize a wide range of lipids from short chain fatty acids (SCFAs) such as acetate, butyrate, and propionate to polyunsaturated fatty acids (PUFAs) involved in regulation of apoptosis and immune response [46, 53–55]. SCFAs are usually synthesized by gut bacteria from nondigestible polysaccharides [56]. Gut microbiome and its metabolites (SCFAs) can affect the immune system by regulating differentiation of resident mucosal immune cells [57]. Previous studies have demonstrated that butyrate can affect the induction of differentiation of colonic regulatory T cells [58]. PUFAs such as punicic and eleostearic acids have also received attention for colitis treatment [59]. The correlation between metabolites and gut bacteria associated with colitis is likely increased from changes in metabolites of microbes [60]. Duncan et al. [61] isolated 9 bacteria strains able to utilize lactate and produce butyrate from fecal samples. The lower quantity of lactate-utilizing butyrate-producing bacteria such as* Clostridium coccoides* could explain the low levels of SCFAs and greater abundance of lactate seen in active colitis [61, 62]. However, it is difficult to identify clear mechanisms involved in the change of metabolites. More precise studies are required to determine the relationship between colonic microorganisms and metabolites.

## Data Availability

The metabolomics data used to support the findings of this study are available from the corresponding author upon request.

## Supplementary Materials

Supplementary Materials Supplementary Figure 1. Chemical structures of the ten marker compounds in Jackyakgamcho-tang. Supplementary Figure 2. Schematic diagram of acute colitis induction and treatment. Supplementary Figure 3. Representative HPLC chromatograms of the (A) standard mixture and (B) Jackyakgamcho-tang sample at UV wavelengths 230 (I), 250 (II), 255 (III), 270 (IV), 275 (V), and 360 (VI) nm. Supplementary Figure 4. RT-PCR analysis of mucosal cytokine expression in acute colitis. Supplementary Figure 5. Representative GC-MS total ion current (TIC) chromatograms of (A) serum and (B) feces samples. Supplementary Figure 6. Microbial community analysis results. Supplementary Table 1. Quality control data of Jackyakgamcho-tang. Supplementary Table 2. Amounts of the ten marker compounds in Jackyakgamcho-tang by HPLC (n=3). Supplementary Table 3. Identified metabolites in serum and feces samples. Supplementary Table 4. Metabolic changes by acetic acid-induced colitis and Jackyakgamcho-tang administration in serum and feces. Supplementary Table 5. Microbial richness and diversity of 16S rRNA libraries based on 97% identity OTUs from airborne bacteria collected with groups, respectively.
