# Supplementary material for: GC/MS-Based Metabolomics Approach to Evaluate the Effect of Jackyakgamcho-Tang on Acute Colitis
Source: Evid Based Complement Alternat Med. 2019 Jan 21;2019:4572764. doi: 10.1155/2019/4572764 (PMC6360583; doi:10.1155/2019/4572764)
Supplement: Supplementary Materials — Supplementary Figure 1. Chemical structures of the ten marker compounds in Jackyakgamcho-tang. Supplementary Figure 2. Schematic diagram of acute colitis induction and treatment. Supplementary Figure 3. Representative HPLC chromatograms of the (A) standard mixture and (B) Jackyakgamcho-tang sample at UV wavelengths 230 (I), 250 (II), 255 (III), 270 (IV), 275 (V), and 360 (VI) nm. Supplementary Figure 4. RT-PCR analysis of mucosal cytokine expression in acute colitis. Supplementary Figure 5. Representative GC-MS total ion current (TIC) chromatograms of (A) serum and (B) feces samples. Supplementary Figure 6. Microbial community analysis results. Supplementary Table 1. Quality control data of Jackyakgamcho-tang. Supplementary Table 2. Amounts of the ten marker compounds in Jackyakgamcho-tang by HPLC (n=3). Supplementary Table 3. Identified metabolites in serum and feces samples. Supplementary Table 4. Metabolic changes by acetic acid-induced colitis and Jackyakgamcho-tang administration in serum and feces. Supplementary Table 5. Microbial richness and diversity of 16S rRNA libraries based on 97% identity OTUs from airborne bacteria collected with groups, respectively. [file 4572764.f1.docx]

Supplementary Materials

**GC/MS-based Metabolomics Approach to Evaluate the Effect of Jackyakgamcho-tang on Acute Colitis**

**Table of contents:**

Supplementary Figure 1: Chemical structures of the ten marker compounds in Jackyakgamcho-tang.

Supplementary Figure 2. Schematic diagram of acute colitis induction and treatment.

Supplementary Figure 3: Representative HPLC chromatograms of the (A) standard mixture and (B) Jackyakgamcho-tang sample at UV wavelengths 230 (I), 250 (II), 255 (III), 270 (IV), 275 (V), and 360 (VI) nm.

Supplementary Figure 4: RT-PCR analysis of mucosal cytokine expression in acute colitis.

Supplementary Figure 5: Representative GC-MS total ion current (TIC) chromatograms of (A) serum and (B) feces samples.

Supplementary Figure 6: Microbial community analysis results.

Supplementary Table 1: Quality control data of Jackyakgamcho-tang.

Supplementary Table 2: Amounts of the ten marker compounds in Jackyakgamcho-tang by HPLC (n=3).

Supplementary Table 3: Identified metabolites in serum and feces samples.

Supplementary Table 4: Metabolic changes by acetic acid-induced colitis and Jackyakgamcho-tang administration in serum and feces.

Supplementary Table 5: Microbial richness and diversity of 16S rRNA libraries based on 97% identity OTUs from airborne bacteria collected with groups, respective.

**Supplementary Figure 1. Chemical structures of the ten major compounds in Jackyakgamcho-tang.**


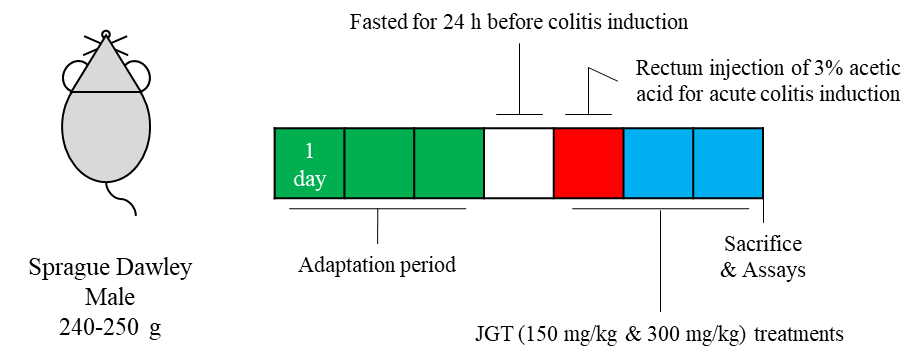


**Supplementary Figure 2. Schematic diagram of acute colitis induction and treatment.**


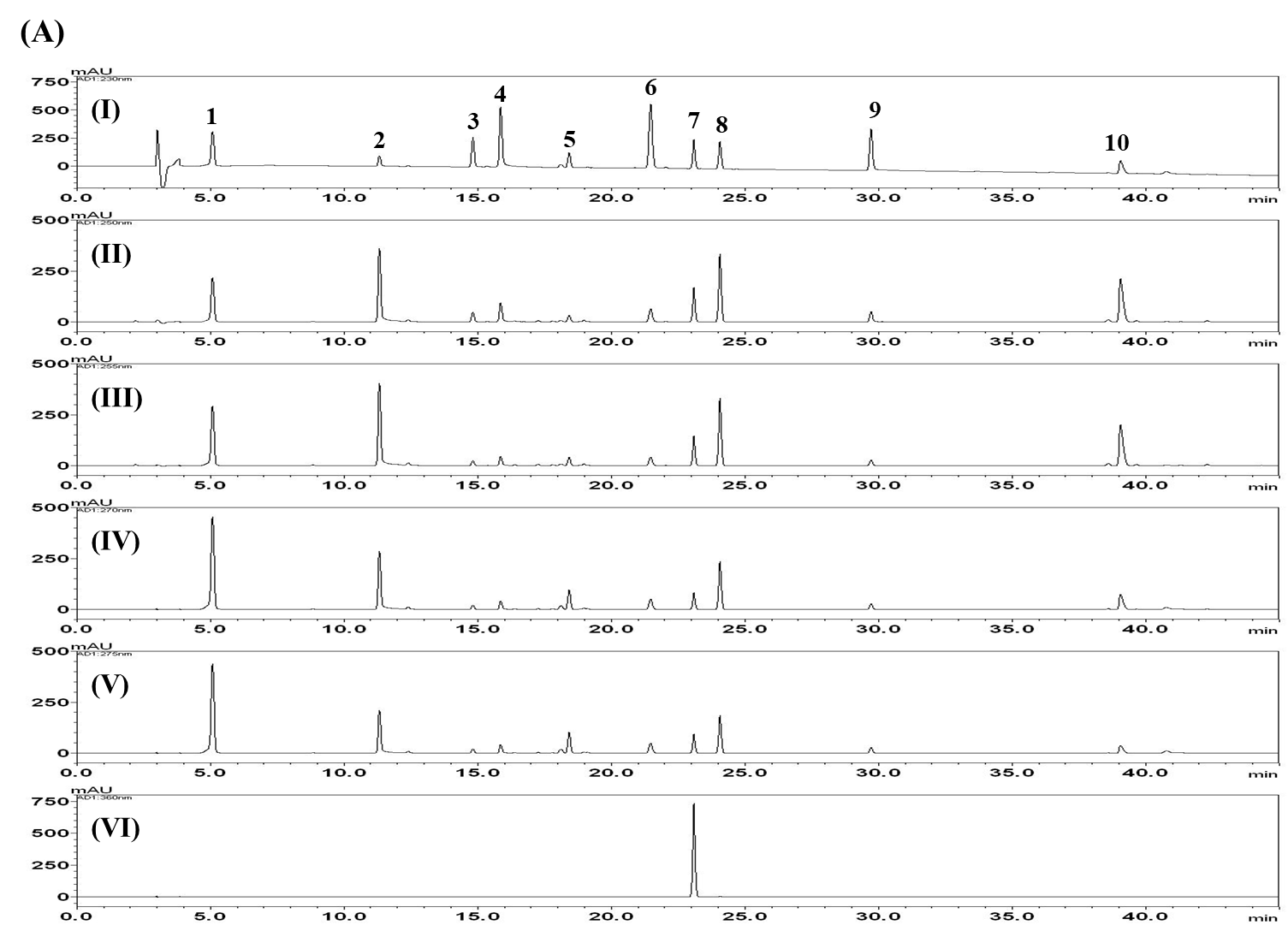

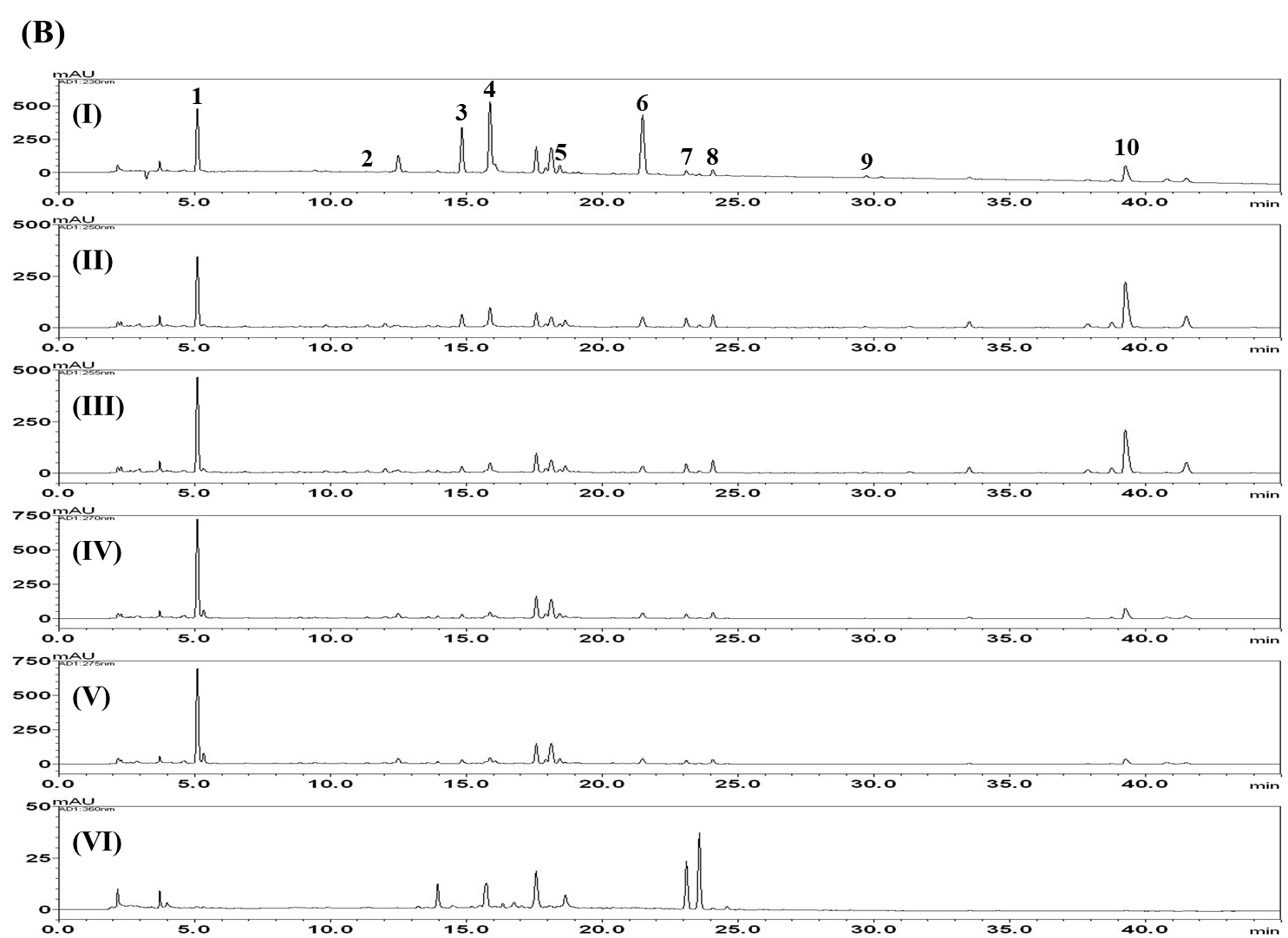


**Supplementary Figure 3. Representative HPLC chromatograms of the (A) standard mixture and (B) Jackyakgamcho-tang sample at UV wavelengths 230 (I), 250 (II), 255 (III), 270 (IV), 275 (V), and 360 (VI) nm.** Gallic acid (1), oxypaeoniflorin (2), albiflorin (3), paeoniflorin (4), liquiritin (5), benzoic acid (6), lsoliquiritin (7), ononin (8), benzoylpaeoniflorin (9), and glycyrrhizin (10).


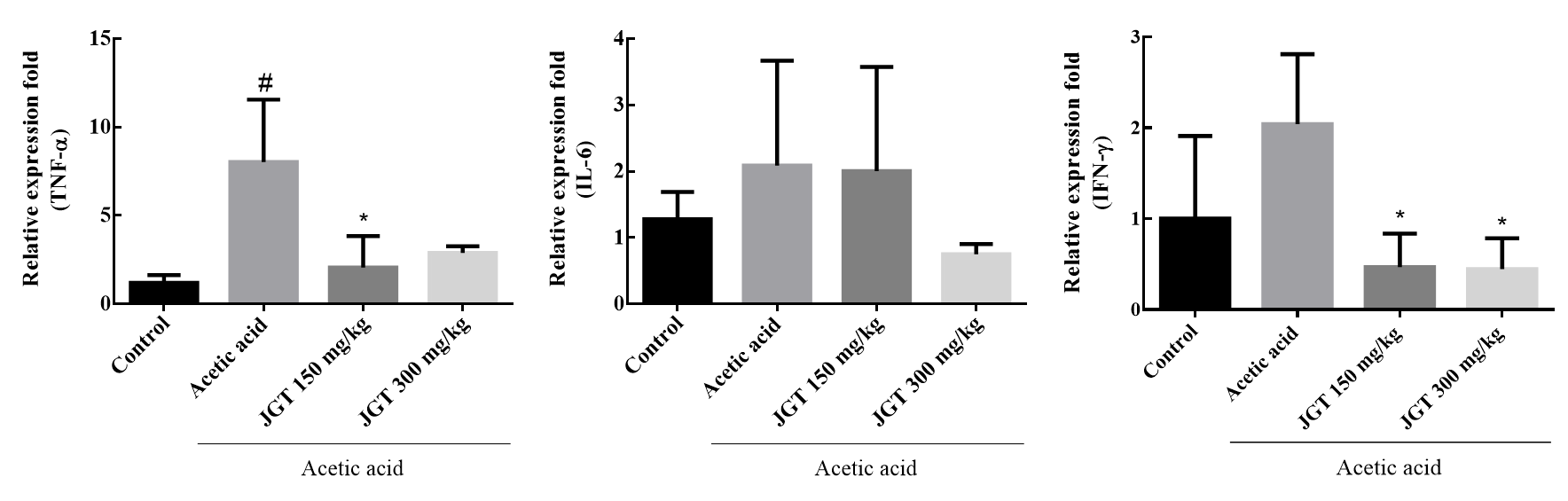


**Supplementary Figure 4. RT-PCR analysis of mucosal cytokine expression in acute colitis.** Significant difference at ^#^*p* < 0.05, ^##^*p* < 0.01, and ^###^*p* < 0.001 compared to the control group. Significant difference at ^*^*p* < 0.05, ^**^*p* < 0.01, and ^***^*p* <0.001 compared to acetic acid-induced acute colitis group.

**
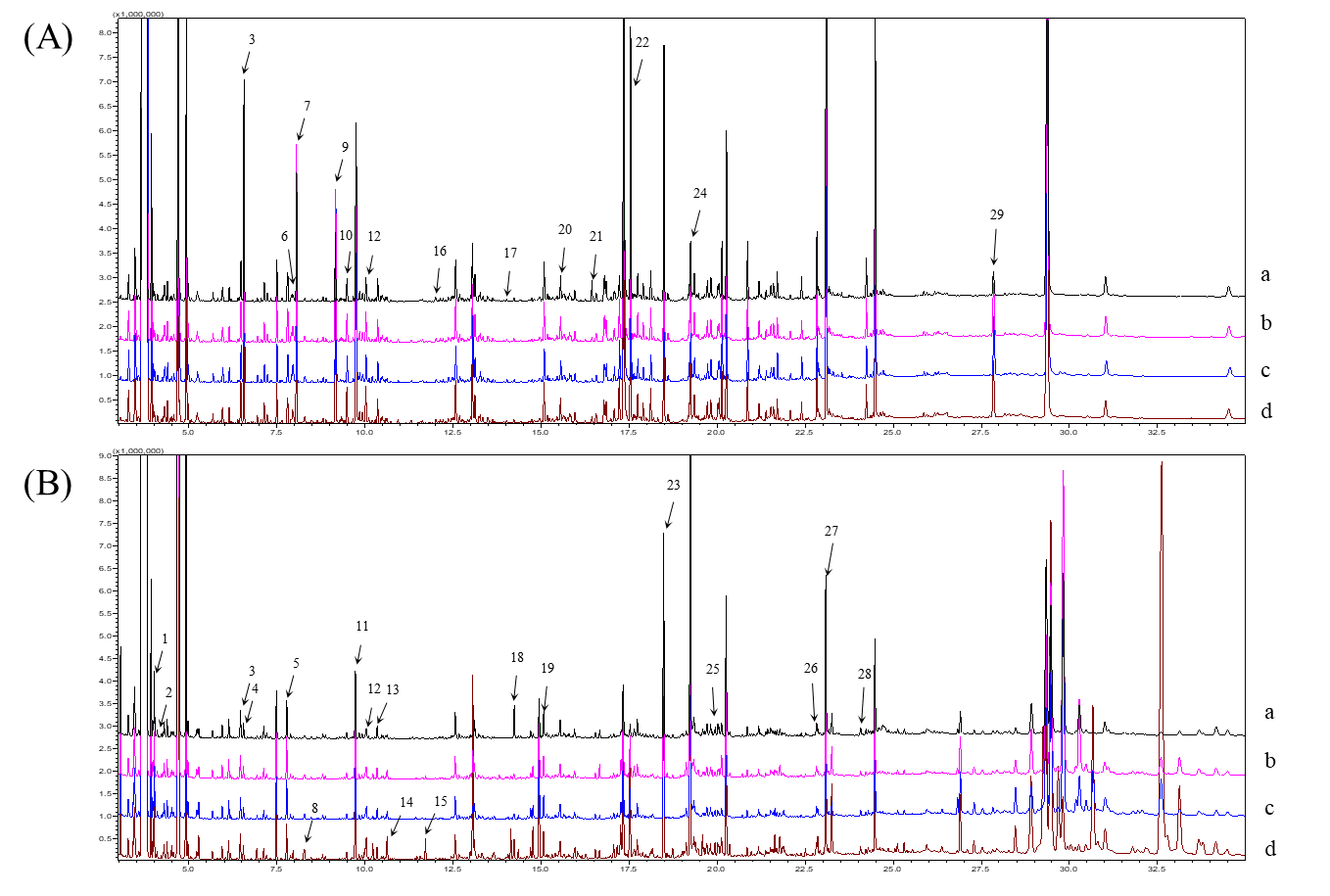
**

**Supplementary Figure 5. Representative GC-MS total ion current (TIC) chromatograms of (A) serum and (B) feces samples.** The ordinate displays the relative mass abundance, and the abscissa displays the retention time. a, Control; b, acetic acid-induced colitis; c, acetic acid-induced colitis+Jackyakgamcho-tang 150 mg/kg treatment; d, acetic acid-induced colitis+Jackyakgamcho-tang 300 mg/kg treatment.

**
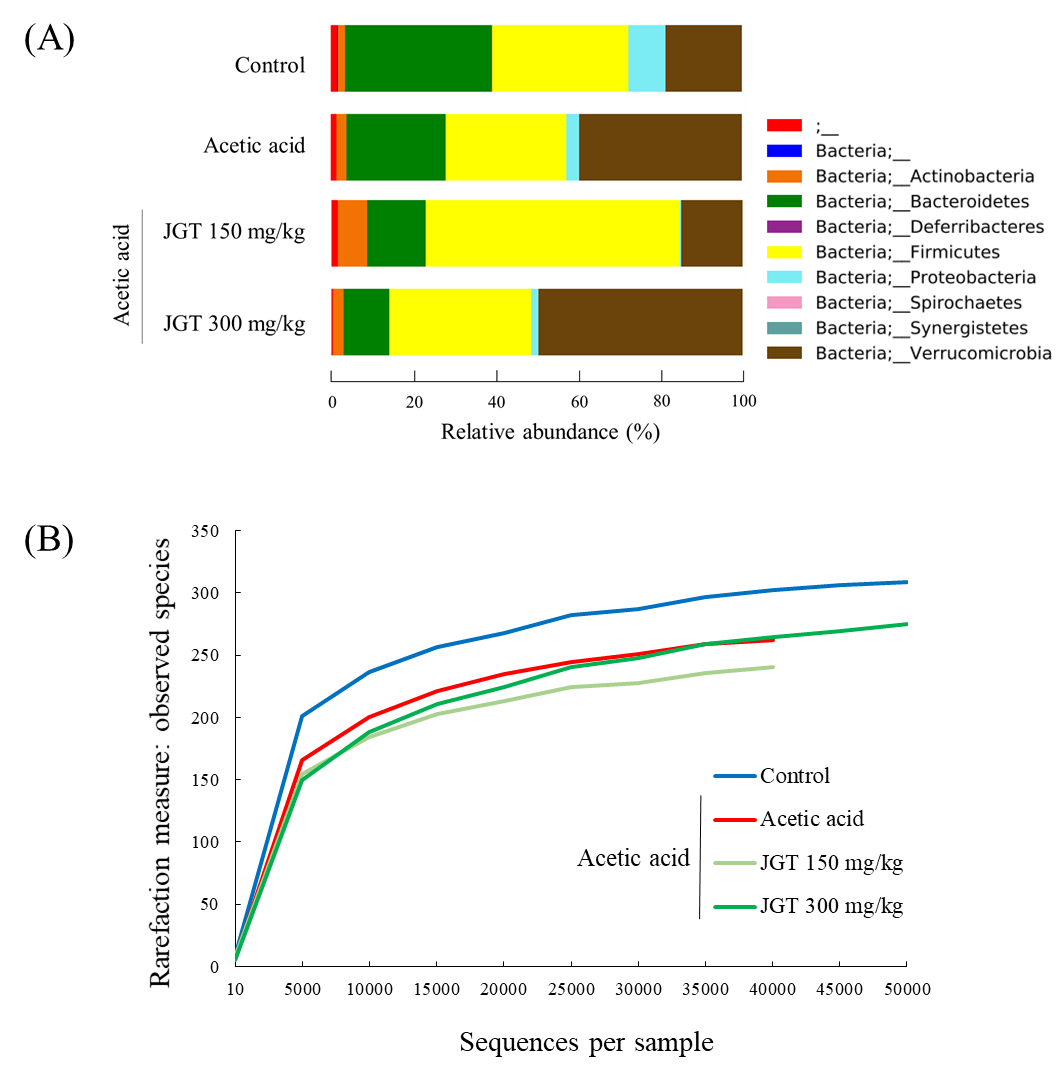
**

Supplementary Figure 6**. Microbial community analysis results.** (A) The gut microbiome composition profiles at the phylum-level in the rats revealed by 16S rRNA sequencing (each color represents one bacterial phylum). (B) Representative rarefaction curves based on the number of OTUs indicated the bacterial diversity within the samples (alpha diversity).

**Supplementary Table 1. Quality control data of Jackyakgamcho-tang**

| Test Name |  | Test standard | Result | Unit |
| --- | --- | --- | --- | --- |
| Appearance |  | Dry matter of yellow-brown | Confirm | - |
| Contents | *Glycyrrhiza* | It should be confirmed. | Confirm | - |
|  | *Paeonia* | It should be confirmed. |  | - |
| Purity test | Heavy metal | Below 30 ppm | N.D. | ppm |
|  | Pesticide residue | Below DDT 0.1 ppm | N.D. | ppm |
|  |  | Below BHC 0.2 ppm | N.D. | ppm |
|  |  | Below Aldrin 0.01 ppm | N.D. | ppm |
|  |  | Below Dieldrin 0.01 ppm | N.D. | ppm |
|  |  | Below Endrin 0.01 ppm | N.D. | ppm |
| Loss on drying |  | Below 5.2% | 3.3% |  |
| Microbial limit test |  | Below aerobe  1ｘ10^5^ | Below 100 | Number |
|  |  | Below fungus  1ｘ10^2^ | Below 10 | Number |
|  |  | *Escherichia coli,*  *Pseudomonas aeruginosa,*  *Salmonella,*  *Staphylococcus aureus*  N.D. | N.D. | - |
| Content | Glycyrrhizic acid content in *Glycyrrhiza* | Above 12.5 | 30.3 | mg/g |
|  | Paeoniflorin content in *Paeonia* | Above 10.8 | 24.7 | mg/g |

**Supplementary Table 2. Amounts of the ten marker compounds in Jackyakgamcho-tang by HPLC (n=3).**

| Compound | Mean (mg/g) | SD^a^ | RSD^b^ | Source |
| --- | --- | --- | --- | --- |
| Gallic acid | 11.24 | 0.124 | 1.11 | *Paeonia lactiflora* |
| Oxypaeoniflorin | 0.98 | 0.005 | 0.48 | *P. lactiflora* |
| Albiflorin | 15.76 | 0.067 | 0.42 | *P. lactiflora* |
| Paeoniflorin | 26.19 | 0.173 | 0.66 | *P. lactiflora* |
| Liquiritin | 1.71 | 0.009 | 0.52 | *Glycyrrhiza uralensis* |
| Benzoic acid | 10.37 | 0.042 | 0.40 | *P. lactiflora* |
| Isoliquiritin | 0.34 | 0.001 | 0.37 | *G. uralensis* |
| Ononin | 1.45 | 0.019 | 1.31 | *G. uralensis* |
| Benzoylpaeoniflorin | 0.63 | 0.004 | 0.67 | *P. lactiflora* |
| Glycyrrhizin | 30.94 | 0.076 | 0.25 | *G. uralensis* |

^a^ SD: Standard deviation (n=3), ^b^ RSD: Relative standard deviation.

**Supplementary Table 3. Identified metabolites in serum and feces samples.**

| **No.** | **RT^a^** | **Metabolites** | **T^b^** | **Q^c^** | **RI^d^** | **ID^e^** | **Similarity(%)** | **Samples** |
| --- | --- | --- | --- | --- | --- | --- | --- | --- |
| 1 | 3.98 | Acetamide | 72 | 87,72 | 881 | RI, MS | 95 | Feces |
| 2 | 4.01 | Butyric acid | 75 | 75,145,73 | 884 | RI, MS | 87 | Feces |
| 3 | 6.54 | Lactica cid | 147 | 147,73,117 | 1063 | RI, MS, R | 83 | Serum, Feces |
| 4 | 6.93 | Valine | 72 | 72,55,75 | 1088 | RI, MS, R | 97 | Feces |
| 5 | 7.88 | Acetic acid | 191 | 73,191,147 | 1152 | RI, MS | 92 | Feces |
| 6 | 7.97 | Leucine | 86 | 86,75,73 | 1159 | RI, MS, R | 93 | Serum |
| 7 | 8.06 | 3-Hydroxybutyric acid | 147 | 147,73,117 | 1165 | RI, MS, R | 96 | Serum |
| 8 | 8.13 | Pentanoic acid | 145 | 73,145,146 | 1169 | RI, MS, R | 76 | Feces |
| 9 | 9.18 | Urea | 147 | 147,189,73 | 1242 | RI, MS, R | 92 | Serum |
| 10 | 9.48 | Serine | 116 | 116,73,132 | 1265 | RI, MS, R | 90 | Serum |
| 11 | 9.72 | Glycerol | 73 | 73,147,205 | 1280 | RI, MS, R | 81 | Feces |
| 12 | 10.01 | Threonine | 117 | 73,117,130 | 1300 | RI, MS, R | 96 | Serum, Feces |
| 13 | 10.22 | Succinic acid | 147 | 147,73,247 | 1317 | RI, MS, R | 94 | Feces |
| 14 | 10.62 | Uracil | 241 | 241,147,99 | 1346 | RI, MS | 93 | Feces |
| 15 | 11.43 | Pentanedioic acid | 147 | 147,73,261 | 1406 | RI, MS | 88 | Feces |
| 16 | 12.37 | L-Hydroxyproline | 158 | 158,68,73 | 1481 | RI, MS | 78 | Serum |
| 17 | 14.03 | Oxoproline | 156 | 156,73,147 | 1620 | RI, MS | 94 | Serum |
| 18 | 14.76 | Pentose(lyxose, xylose) | 73 | 73,103,217 | 1683 | RI, MS, R | 93 | Feces |
| 19 | 15.35 | Propanoic acid | 192 | 192,205,73 | 1736 | RI, MS, R | 86 | Feces |
| 20 | 15.84 | Phosphoric acid | 299 | 73,299,357 | 1783 | RI, MS | 74 | Serum |
| 21 | 16.81 | 1,5-Anhydrohexitol | 73 | 73,147,217 | 1876 | RI, MS | 94 | Serum |
| 22 | 17.54 | Pyranose(talose, allose, glucose, galactose) | 319 | 73,319,147 | 1950 | RI, MS, R | 93 | Serum |
| 23 | 18.16 | Pantothenic acid | 157 | 73,103,117 | 2012 | RI, MS | 76 | Feces |
| 24 | 19.21 | Myo-inositol | 217 | 73,305,217 | 2126 | RI, MS, R | 83 | Serum |
| 25 | 20.01 | Linoleic acid | 67 | 73,75,67 | 2215 | RI, MS, R | 88 | Feces |
| 26 | 22.82 | Palmitoyl glycerol | 129 | 129,73,218 | 2560 | RI, MS | 89 | Feces |
| 27 | 23.08 | Palmitic acid | 147 | 371,147,73 | 2595 | RI, MS | 88 | Feces |
| 28 | 24.07 | Monostearin | 129 | 129,103,73 | 2730 | RI, MS | 81 | Feces |
| 29 | 27.85 | Cholesterol | 133 | 129,329,73 | 3305 | RI, MS, R | 93 | Serum |

^a^ RT, retention time; ^b^ T, target ion; ^c^ Q, qualifier ions; ^d^ RI, retention index (NIST v14.0); ^e^ ID, identification; ^f^ MS, mass spectrum of NIST 14; ^g^ R, retention time and qualifier ions of reference paper (Mastrangelo et al. 2015)

**Supplementary Table 4. Metabolic changes by acetic acid-induced colitis and Jackyakgamcho-tang administration in serum and feces.**

| Collect | Metabolites | Acetic acid-induced colitis  vs.  Control | | | JGT+Acetic acid induced colitis  vs.  Acetic acid-induced colitis | | |
| --- | --- | --- | --- | --- | --- | --- | --- |
|  |  | Fold change | ↑/↓^a^ | *p*-value | Fold change | ↑/↓ | *p*-value |
| Serum | Lactic acid | 1.16 | ↑ | 0.064 | 1.25 | ↓ | 0.034 |
| Feces | Linoleic acid | 1.83 | ↓ | 0.041 | 1.47 | ↑ | 0.067 |
|  | Monostearin | 1.7 | ↓ | 0.015 | 1.43 | ↑ | 0.003 |
|  | Palmitoylglycerol | 1.37 | ↓ | 0.034 | 1.65 | ↑ | 0.002 |

^a^ The arrows (↑ and ↓) represent a decrease or increase in the metabolite levels in acetic acid-induced colitis compared to the control group and in Jackyakgamcho-tang (150 mg/kg and 300 mg/kg) + acetic acid-induced group compared to the acetic acid colitis group. The levels were estimated from the relative intensities of the GC/MS spectra of serum and fecal extracts following spectral normalization. Metabolites above *p* = 0.10 are not indicated.

**Supplementary Table 5. Microbial richness and diversity of 16S rRNA libraries based on 97% identity OTUs from airborne bacteria collected with control, acetic acid-induced acute colitis, and Jackyakgamcho-tang treatment (150 mg/kg and 300 mg/kg injection) acute colitis groups.**

| Sample | | Chao^a^ | Shannon^b^ | Simpson^c^ | Goods coverage^d^ |
| --- | --- | --- | --- | --- | --- |
| Control | | 327.85 | 5.54 | 0.94 | 0.99 |
| Acetic acid injection | Acetic acid | 299.91 | 4.30 | 0.83 | 0.99 |
|  | Jackyakgamcho-tang 150 mg/kg | 271.95 | 4.47 | 0.92 | 0.99 |
|  | Jackyakgamcho-tang 300 mg/kg | 298.50 | 3.65 | 0.74 | 0.99 |

^a^ Chao1: returns the Chao1 richness estimate for an OTU definition.

^b^ Shannon: The Shannon index takes into account the number and evenness of species.

^C^ Simpson: The Simpson index represents the probability that two randomly selected individuals in the habitat will belong to the same species.

^d^ Goods coverage: Coverage is calculated as C=1-(s/n), where s is the number of unique OTUs and n is the number of individuals in the sample. This index gives a relative measure of how well the sample represents the larger environment.

Supplementary Materials Reference

Mastrangelo A, Ferrarini A, Rey-Stolle F, García A, Barbas C. 2015. From sample treatment to biomarker discovery: a tutorial for untargeted metabolomics based on GC-(EI)-Q-MS. Anal Chim Acta. 900:21-35.
